# Combined Effect of Entomopathogens against *Thrips tabaci* Lindeman (Thysanoptera: Thripidae): Laboratory, Greenhouse and Field Trials

**DOI:** 10.3390/insects12050456

**Published:** 2021-05-16

**Authors:** Sehrish Gulzar, Waqas Wakil, David I. Shapiro-Ilan

**Affiliations:** 1 Department of Entomology, University of Agriculture, Faisalabad 38040, Pakistan; sehrishgulzar41@gmail.com; 2 SE Fruit & Tree Nut Research Laboratory, USDA-ARS, Byron, GA 31008, USA

**Keywords:** *Heterorhabditis bacteriophora*, *Steinernema carpocapsae*, *Beauveria bassiana*, *Metarhizium anisopliae*, *Thrips tabaci*, combined effects, interaction, developmental stage

## Abstract

**Simple Summary:**

Onion thrips *Thrips tabaci* Lindeman (Thysanoptera: Thripidae) is one of the most damaging insect pests of onions, *Allium cepa* L., which is an economically important agricultural crop cultivated worldwide. In the present study, the combination of *Heterorhabditis bacteriophora* (VS strain), *Steinernema feltiae* (SN strain) with *Beauveria bassiana* (WG-11) and *Metarhizium anisopliae* (WG-02) caused greater mortality compared to lone application of each agent, with prominent additive interaction observed. The pre-pupal stage was found to be the most susceptible stage compared to pupae and late second instar larvae of *T. tabaci*. In potted plant bioassay under greenhouse conditions, again, combination of pathogens produced significantly fewer adults compared to lone application of each pathogen. In field trials, combination of pathogens showed lower numbers of larvae and adults compared to lone application of each pathogen, and an increase in plant growth was also observed among the treated group compared to the control group.

**Abstract:**

Onion thrips, *Thrips tabaci* Lindeman (Thysanoptera: Thripidae) is one of the most damaging insect pests of onions, *Allium cepa* L., which is an economically important agricultural crop cultivated worldwide. In this study, the combined application of entomopathogenic nematodes with entomopathogenic fungi against different soil dwelling stages of *T. tabaci* was evaluated. The nematodes included *Heterorhabditis bacteriophora* (VS strain) and *Steinernema feltiae* (SN strain), and fungi included *Beauveria bassiana* (WG-11) and *Metarhizium anisopliae* (WG-02); all four paired combinations (nematode + fungus) were included. In a small cup bioassay, only the combined application of *H. bacteriophora* and *B. bassiana* (WG-11) caused a synergistic interaction against pre-pupae, while all other combinations were compatible in an additive manner against pupae and late second instars. In a larger arena, a potted soil bioassay, again, combined applications of both pathogens produced greater mortality compared to single applications of each pathogen; all the combinations exhibited additive interactions, with the highest mortality observed in pre-pupae, followed by pupae and late second instar larvae using *H. bacteriophora* and *B. bassiana* (WG-11). Additionally, in the potted plant bioassay, lower adult emergence was observed from treated groups compared to control groups. Under field conditions, lower numbers of adults and larvae were found in treated groups relative to controls. Overall, the pre-pupal stage was more susceptible to the pathogen treatments, followed by pupae and late second instar larvae, and also combined applications of both pathogens suppressed the adult population. Combined application of entomopathogenic nematodes and fungi could be used for integrated pest management (IPM) of *T. tabaci* in onion production systems.

## 1. Introduction

Onion thrips, *Thrips tabaci* Lindeman (Thysanoptera: Thripidae), is a serious polyphagous pest of vegetable crops causing huge damage throughout the world [1]. It is a severe pest of *Alliaceae* plants, i.e., onions, leek [2], and *Brassicaceae* plants, like cabbage [3]. Mostly damage is produced by larvae and adult stages, as they feed on green leaves, causing direct losses by abolishing the plant epidermal cells. They pierce the leaf surface and suck the sap contents from leaf tissues [4]. Infested plants display silvery white spots resulting in cosmetic damage that decrease plant marketability. *Thrips tabaci* also serve as a vector of various plant viruses, including Iris yellow spot virus [5,6], tobacco streak virus [7], sowbane mosaic virus [8], and tomato spotted wilt virus [9,10].

The thrips life cycle is comprised of first and second instar larvae, and adults (foliar feeders), as well as late second instar larvae, pre-pupae, and pupae (soil inhabiting stages). Thrips move to soil at the late second instar stage where they molt into pre-pupae and pupae [11]. Thus, an ideal control technique would be to target both developmental stages (foliar and soil living) of the pest [12]. Pre-pupae and pupae are immobile soil living stages and mostly susceptible to soil inhabiting pathogens [12,13,14].

Commonly, *T. tabaci* is targeted with the application of synthetic insecticides, which results in the presence of insecticide residues in food products, resistance development, and can be costly [15,16,17,18,19]. Due to increasing concern of repeated, injudicious, and overuse of insecticides, growers are looking for more eco-friendly approaches to manage the insect pest, such as biological control approaches. Within biological control agents, some predators, such as predatory mites (*Amblyseius* spp.) and Hemiptera (*Orius* spp.), have been reported [20]. However, efficacy of these predators has been limited [21].

Entomopathogenic nematodes (EPNs) have also become progressively accepted over the last 2–3 decades as a biological control tool, particularly against soil dwelling pests [22,23]. EPNs find their hosts by ambusher, cruiser, or intermediate foraging behaviors [24]. The lifecycle of EPN consists of an egg, four infective juvenile stages (IJs), and adults. The nematodes kill their hosts with the aid of mutualistic bacterial symbionts. EPNs in the genus *Steinernema* are associated with *Xenorhabdus* spp. bacterial symbionts, and *Heterorhabditis* spp. EPNs are associated with *Photorhabdus* spp. bacteria [25]. Infective juveniles (IJs) penetrate into the host body via the anus, mouth, cuticle, or spiracles, followed by the release of their bacteria, and, subsequently, the insect dies from septicemia or toxemia within 48 h [26,27]. EPNs are safe to humans and other vertebrates, and have little or no harmful effects on other non-targets [28]. Earlier studies reported that EPNs had a great effect on *T. tabaci* [29,30] and Western flower thrips *Frankliniella occidentalis* Pergande (Thysanoptera: Thripidae) [31]. There is a dearth of literature on the efficacy of EPNs against *T. tabaci.* Additionally, EPNs proved less effective against foliar stages (first and second instar larvae) and exhibited significant potential to control soil inhabiting stages (late second instar, pre-pupae and pupae) [29,30,32].

Another group of biological control agents, entomopathogenic fungi (EPF), has also shown promise as an alternative to insecticides for thrips management [33]. Fungal infection starts with attachment of conidia to the insect cuticle, followed by penetration into the insect hemocoel, where proliferation occurs [34,35]. For successful infection, EPF produce various enzymes (chitinases, lipases, and proteases) which enable adherence, as well as penetration of spores throughout the cuticle [36]. After penetration, spores germinate, reproduce, and eventually kill the insects. Literature on the efficacy of EPF as a potential biocontrol tool against *T. tabaci* is scarce [37,38,39].

Application of entomopathogens has not consistently resulted in sufficient economic control of thrips [40,41]. Consequently, using a set of insect pathogens [42,43] that could have positive effects (synergistic and additive) in efficacy may offer a sustainable approach in insect management systems. Combined use of EPNs and EPF was documented to provide increases in efficacy by [44,45,46,47,48,49] against other insect pests. Otieno et al. [50] evaluated the combined application of EPN and EPF to manage *F. occidentalis*. To the best of our knowledge, no previous study investigated the combined efficacy of EPF and EPNs in controlling *T. tabaci*. Thus, our objective was to explore the efficacy of EPNs and EPF in dual applications against *T. tabaci* under laboratory, greenhouse, and field conditions.

## 2. Materials and Methods

### 2.1. Insect Rearing

A laboratory population of *T. tabaci* was started with field collected adults from University of Agriculture, Faisalabad, Punjab, Pakistan. Onion thrips were constantly reared by providing fresh cabbage leaves in large Petri plates (150 mm in diameter). A small Petri plate (60 mm in diameter) was used as a water reservoir, and a cut at the side wall of this small plate was made for insertion of cabbage leaves; the small dish was attached to the bottom of the large plate. A fresh cabbage leaf was placed into dry filter paper in the bottom of the large plate and a petiole of this leaf inserted in the reservoir, enclosed with a saturated cotton pad with distilled water and covered with the plate lid. Fifteen to twenty adult thrips (female) were released on a cabbage leaf in the large plate and covered with a lid. The large plate lid contained a fine sieve at the center of the plate for ventilation. The plates were placed in an incubator at 25 °C and with a 16:8 h (light:dark) photoperiod. Water in the reservoir plate was refilled on a daily basis to maintain moisture levels. After 3–4 days, thrips were transferred onto new fresh cabbage leaves.

### 2.2. Entomopathogenic Nematodes

The EPNs *Heterorhabditis bacteriophora* (VS strain) and *Steinernema feltiae* (Filipjev) (SN strain) used during the present study were obtained from Microbial Control Laboratory, Department of Entomology, University of Agriculture Faisalabad; originally, these species were obtained from the USDA-ARS collection in Byron, Georgia, USA. The EPNs were cultured on last instar larvae of the greater wax moth *Galleria mellonella* L. (Lepidoptera: Pyralidae) and IJs were collected on white traps [51]. The EPNs were stored in tissue culture flasks (250 mL) incubated at 14 °C. EPNs were less than two weeks when used in bioassays.

### 2.3. Entomopathogenic Fungi

Entomopathogenic fungi (EPF) *Beauveria bassiana* (Bals.-Criv.) Vuill. (Hypocreales: Cordycipitaceae) (WG-11) and *Metarhizium anisopliae* (Metchnikoff) Sorokin (Hypocreales: Clavicipitaceae) (WG-02) were taken from the culture collection of Microbial Control Laboratory, Department of Entomology, University of Agriculture Faisalabad. Each isolate was individually cultured on Potato Dextrose Agar (PDA) Petri plates (100 mm) that were wrapped with parafilm and incubated at 25 °C with a 14:10 h (light:dark) photoperiod. Fungi were scraped from inoculated plates 7–10 days post-incubation with the help of a sterile scalpel, and the resulting conidia were put inside conical tubes (50 mL) that contained 30 mL of 0.05% Silwet L-77 solution. Eight glass beads were added inside each tube and vortexed for about 5 min, and then the desired concentration (1 × 10^7^ conidia mL^−1^) was determined using a hemocytometer under the microscope. Conidial viability of each isolate was assessed by plating 0.1 mL of solution of each of the two isolates on small Sabouraud Dextrose Agar Yeast (SDAY) Petri plates (60 mm) [52], followed by incubation at 25 °C with a 14:10 h (light:dark) photoperiod. Germination (%) was determined under the microscope after 16 h post-incubation by putting a cover slip on plates and a total of 200 spores were assessed from each plate. A total of four counts (two counts from each plate) were taken per fungal isolate and then the required concentration was adjusted according to germination (%) of each isolate [49].

### 2.4. Small Cup Bioassay in Laboratory

The aim of this bioassay was to assess the virulence of *B. bassiana* (WG-11), *M. anisopliae* (WG-02), *H. bacteriophora*, and *S. feltiae* alone and in combination against late second instar larvae, pre-pupae, and pupae of *T. tabaci* in small cups. The bioassay was conducted at the Microbial Control Laboratory, Department of Entomology, University of Agriculture Faisalabad using a completely randomized design (CRD). The bioassay arena consisted of 30 mL transparent plastic cups (diameter 10 cm^2^) that were filled with 20 g of sterile sandy loam soil (57% sand, 25% silt, 18% clay, pH 7.6, organic matter 0.95%) and an initial moisture content of 0%. The bioassay consisted of eight treatments plus a control group including applications of each agent alone, *H. bacteriophora*, *S. feltiae* (both at 50 IJs cm^−2^), *B. bassiana* (WG-11) *M. anisopliae* (WG-02) (both at 1 × 10^7^ conidia mL^−1^), and their combinations, *H. bacteriophora* + *B. bassiana* (WG-11), *H. bacteriophora* + *M. anisopliae* (WG-02), *S. feltiae* + *B. bassiana* (WG-11), *S. feltiae* + *M. anisopliae* (WG-02), plus the untreated control group. For single-treatment application of EPNs, 1 mL containing 500 IJs mL^−1^ (50 IJs cm^−2^) was applied to the surface of the soil, followed by the addition of 1 mL of distilled water to maintain soil moisture content at 10%. Subsequently, ten individuals of different developmental stages, i.e., late second instar larvae, pre-pupae, and pupae, were released on the top of the soil. For single-treatment application of EPF, 1 mL of each EPF isolate (1 × 10^7^ conidia mL^−1^) and 1 mL of distilled water was pipetted on the top of the soil and then mixed thoroughly to ensure the equal distribution of fungi throughout the soil, and, subsequently, different developmental stages of *T. tabaci* were released on the top of the soil. For combined applications, first 1 mL of EPF (1 × 10^7^ conidia mL^−1^) was applied, agitated, and then 1 mL containing 500 IJs mL^−1^ (50 IJs cm^−2^) was applied on the top surface of the soil, followed by the release of different developmental thrips stages onto the soil surface. The control group only received 2 mL of distilled water without the addition of conidia or IJs. Cup lids were lined with yellow sticky traps to capture the emerging adults. The lidded cups were placed on trays with wet paper towel to retain moisture inside the cups and incubated at 25 °C with a 14:10 h (light:dark) photoperiod. Seven days post-application the total numbers of emerged adults were observed on the soil, and also on the sticky traps, under a stereomicroscope. Emerged adults were counted as live while non emerged adults were considered to be dead. Each treatment consisted of three replicates with 10 individual per cup per replicate, and the entire experiment was repeated a second time with new individuals (total of 60 insects for each treatment) [49].

### 2.5. Potted Soil Bioassay in Laboratory

The aim of this bioassay was to investigate combined applications of EPF and EPNs against various stages of *T. tabaci* in a larger arena than in the prior assay using completely randomized design (CRD). The bioassay arena consisted of 50 cm diameter plastic pots filled with approximately 200 g of sterile sandy loam soil (57% sand, 25% silt, 18% clay, pH 7.6, organic matter 0.95%) with an initial moisture content of 0%. The treatments were the same as in the previous experiment. For single applications of EPNs, 19 mL of water was applied first, followed by 1 mL containing 2500 IJs mL^−1^ (equal to 50 IJs cm^−2^) applied to the soil surface; soil was agitated for equal distribution. Subsequently, 20 thrips of the different developmental stages (late second instar larvae, pre-pupae, or pupae) were released onto the soil surface. For single applications of EPF, 13 mL of distilled water and 7 mL (1 × 10^7^ conidia mL^−1^) of each EPF isolate were pipetted on the soil surface, and the soil was agitated thoroughly to ensure the equal distribution of fungi throughout the soil. Insects were then added as described above. For combined application, the first 7 mL of EPF (1 × 10^7^ conidia mL^−1^) was applied, 12 mL of water was added and soil agitated for uniform distribution, then 1 mL containing 2500 IJs mL^−1^ (equal to 50 IJs cm^−2^) was applied on the soil surface; insects were applied as described above. The control group only received 20 mL of distilled water without the addition of EPNs or EPF. Petri plate (100 mm) covers lined with yellow sticky traps were placed on the top of the plastic pots. Pots were placed onto the trays and bagged with wet paper towel to retain the moisture content and incubated at 25 °C with a 14:10 h (light:dark) photoperiod. Each pot was a replicate with 20 individuals of each stage, and each treatment consisted of three replicates (three pots). The entire experiment was conducted two times. Adult emergence was determined as described previously at 7 days post-application of treatment [49].

### 2.6. Potted Plant Bioassay in Greenhouse

The objective of this bioassay was to assess the efficacy of different treatments against the soil-dwelling stages of *T. tabaci* under greenhouse conditions using randomized complete block design (RCBD). Plastic pots were planted with onion seeds (desi red variety), and individual pots were placed inside cages (30 × 30 × 30 cm) that contained thrips-proof netting for ventilation. At the 3–5 leaves stage, the pots were infested with 10 female and 2 male adults using a fine camel hairbrush. The thrips were allowed to mate and fly for 72 h. Prior to insect release on the plant, the adults were cold anaesthetized at 4 °C for 20 min to slow down their activity for easy handling. After the fixed time intervals, the plants were shaken well and all the adults were collected on white paper. Eight days post insect release, the different treatments were applied. The bioassay consisted of the same eight treatments described above and a control. For single treatment EPN applications, 1 mL containing 2500 IJs mL^−1^ (50 IJs cm^−2^) was drenched evenly on top of the soil and, five min after the treatment, 19 mL of distilled water was evenly applied throughout the soil. For EPF applied alone, 7 mL of solution was pipetted on top of the soil, and then 13 mL of water was applied throughout the soil to ensure the field capacity on top of the soil. For combined applications, 7 mL (1 × 10^7^ conidia mL^−1^) was pipetted on top of the soil, 1 mL containing 2500 IJs mL^−1^ (equal to 50 IJs cm^−2^) was drenched thoroughly, and then 12 mL distilled water was added to reach field capacity. Controls only received the same amount of distilled water without the addition of EPNs or EPF. Twelve days after the insects were released, the plants were cut from the base. On the roof of cages, the yellow sticky traps were hung with a small white ribbon just on the top of the pots to capture the emerging adults. Beginning seven days after treatment, daily emerging adults were counted on the yellow sticky traps for seven additional days. Each treatment consisted of three replicates, and whole the experiment was repeated twice [50].

### 2.7. Field Trials

Onion cultivar (red desi) was transplanted during December 2017–2018 and 2018–2019 in a 10 m^2^ plot. The distance between the plots within each block was maintained at 70 cm. The distance between plants was 10 cm, and between rows the distance was 30 cm. All the recommended nutrients were applied and weeds were removed manually by hand. The plots were watered when necessary with flood irrigation. The experiment consisted of eight treatments, including single applications of each agent *H. bacteriophora*, *S. feltiae*, *B. bassiana* (WG-11), *M. anisopliae* (WG-02), and the four nematode–fungus combinations, *H. bacteriophora* + *B. bassiana* (WG-11), *H. bacteriophora* + *M. anisopliae* (WG-02), *S. feltiae* + *B. bassiana* (WG-11), *S. feltiae* + *M. anisopliae* (WG-02), as well as a non-treated control. The EPNs were applied at 2.5 billion ha^−1^ and treatments were arranged in randomized complete block design (RCBD) with six replicates. The EPNs were applied with a knapsack sprayer. The fungi were suspended in 0.05% of Silwet L-77 solution and applied with a knapsack sprayer at rate 1 × 10^8^ conidia ha^−1^. The control group only received distilled water. The application was conducted at sunset to avoid damage from ultraviolet radiation. Assessments were made 1 day before application (DBA) and then 3, 7, 11, and 15 days post-application (DPA). For assessments, ten plants were randomly selected from different points within the plot (treatment) and insects inspected visually. Moreover, the effects of different treatments on plant growth were determined by uprooting ten randomly selected plants from different points of each plot and recording leaf length with a measuring tape, neck and bulb diameter were recorded with a sliding caliper scale, number of leaves and bulb rings were counted manually, and leaf weight was measured using a weight balance (ATX 224, Shimadzu Corporation, Kyoto, Japan). For bulb weight, dry matter and yield per plant, the plant components were placed under shade for three weeks and then measured for weight.

### 2.8. Statistical Analysis

Mortality data were generated on the basis of adult emergence within each insect stage. Mortality data were corrected by using Abbott’s formula [53] and subjected to analysis of variance (ANOVA), and their mean was compared using Tukey’s HSD test at 5% significance level [54] in Minitab [55]. The interaction (additive, synergism, or antagonistic) was determined between the fungi and nematode on the basis of comparing observed mortality versus expected mortality [56]. The formula *PE* = *P*0 + (1 − *P*0) (*P*1) + (1 − *P*0) (1 − *P*1) (*P*2) was used to determine the expected mortality, where *PE* is the expected mortality of both pathogens, *P*0 mortality in the control treatment, *P*1 mortality from one pathogen, and *P*2 mortality from the second pathogen. A chi-square formula was then applied to compare the observed versus expected results: *X*^2^ = (*L*0 − *LE*)^2^/*LE* + (*D*0 − *DE*)^2^/*DE*, where *L*0 is the number of living individuals observed from treatment, *LE* expected living individuals from treatment, *D*0 number of dead individuals observed from treatment, and *DE* expected dead individuals from treatment. Interactions were additive if *X*^2^ < 3:84, antagonistic if *X*^2^ > 3:84 and *PC* < *PE*, and synergistic if *X*^2^ > 3:84 and *PC* > *PE*, where *PC* is the observed mortality from the combination and *PE* is the expected mortality from the combination. For greenhouse and field bioassays, the data were analyzed with ANOVA under a randomized complete block design (RCBD). Means were separated using Tukey’s test and differences were considered significant if *p* < 0.05.

## 3. Results

### 3.1. Small Cup Bioassay in Laboratory

**Figure 1:**
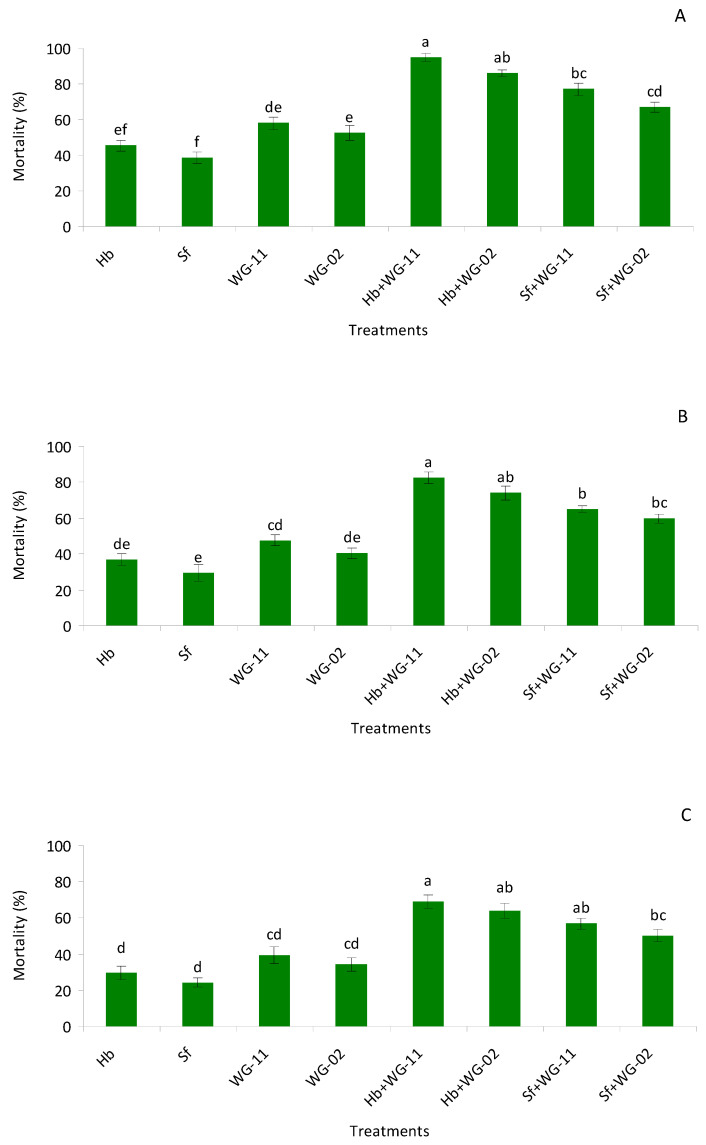
Mean (% ± SE) mortality of pre-pupae (**A**), pupae (**B**) and late last instar larvae (**C**) of *Thrips tabaci* when treated with Hb = *Heterorhabditis bacteriophora*, Sf = *Steinernema feltiae*, WG-11 = *Beauveria bassiana* (WG-11), WG-02 = *Metarhizium anisopliae* (WG-02), Hb + WG-11 = *H. bacteriophora* + *B. bassiana*, Hb + WG-02 = *H. bacteriophora* + *M. anisopliae*, Sf + WG-11 = *S. feltiae* + *B. bassiana*, Sf + WG-02 = *S. feltiae* + *M. anisopliae* in a laboratory small cup bioassay. Different letters above the bars indicate statistical significance (*p* < 0.05; Tukey’s test).

In pre-pupae, a significant difference (*F* = 44.2, df = 7, 47, *p* < 0.01) was detected among the different treatments, yet single-application treatments caused lower mortality compared to combined applications. In single applications, EPF was found to be more virulent compared to EPNs. The highest mortality was observed in the combined application of *H. bacteriophora* + *B. bassiana* (WG-11), which exhibited a synergistic interaction, whereas the rest of the combinations had additive interactions (Figure 1A–C).

The combination treatments were not statistically different from each other. In pupae, a significant difference (*F* = 57.8, df = 7, 47, *p* < 0.01) was observed between single and combined treatments. All combinations exhibited additive interactions, and no differences were observed between combination and single applications of each agent. In late second instars, significantly lower mortality was observed in single applications compared to combination treatments (*F* = 70.0, df = 7, 47, *p* < 0.01). Again, additive interactions were observed among all the combinations and no differences were observed among combination treatments (Figure 1A–C; Table 1).

### 3.2. Potted Soil Bioassay in Laboratory

**Figure 2:**
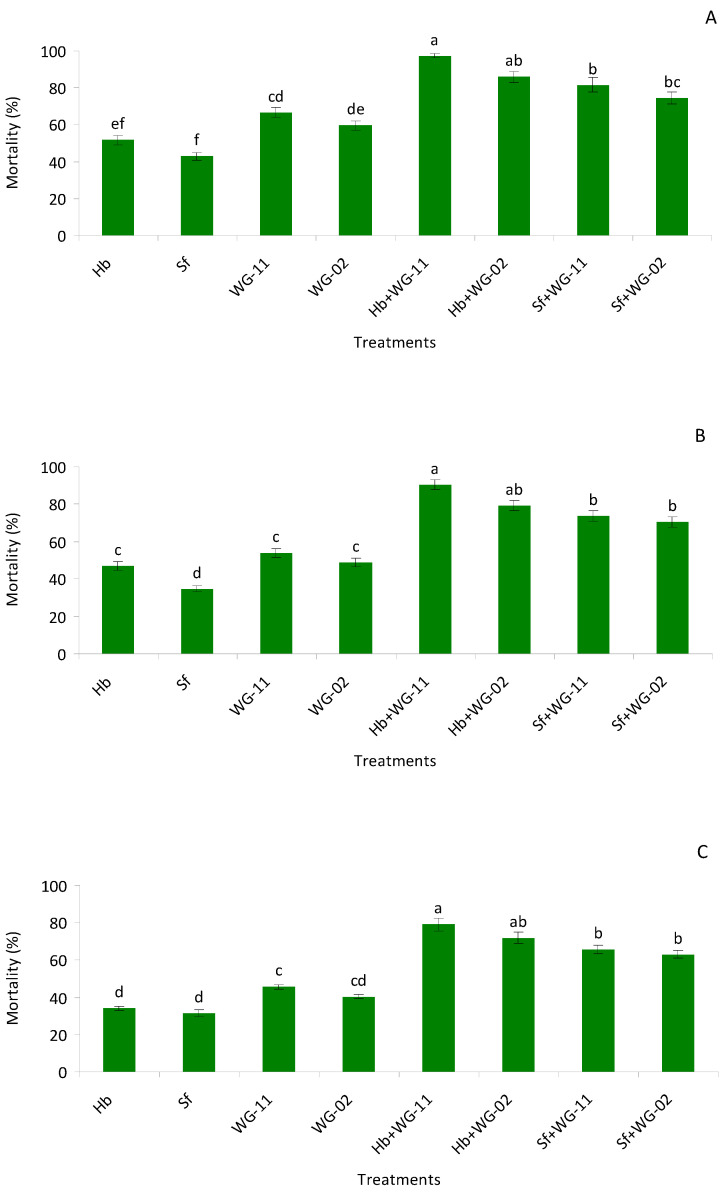
Mean (% ± SE) mortality of pre-pupae (**A**), pupae (**B**) and late last instar larvae (**C**) of *Thrips tabaci* when treated with Hb = *Heterorhabditis bacteriophora*, Sf = *Steinernema feltiae*, WG-11 = *Beauveia bassiana* (WG-11), WG-02 = *Metarhizium anisopliae* (WG-02), Hb + WG-11 = *H. bacteriophora* + *B. bassiana*, Hb + WG-02 = *H. bacteriophora* + *M. anisopliae*, Sf + WG-11 = *S. feltiae* + *B. bassiana*, Sf + WG-02 = *S. feltiae* + *M. anisopliae* in a laboratory potted soil bioassay. Different letters above the bars indicate statistical significance (*p* < 0.05; Tukey’s test).

In potted soil bioassay, significant higher mortality was observed among the different treatments compared to the control group (*F* = 41.0, df = 7, 47, *p* < 0.01) in the pre-pupae stage. Similar to the small cup bioassay, among single treatment applications, *B. bassiana* (WG-11) caused the highest mortality, whereas, for combination treatments, *H. bacteriophora* + *B. bassiana* (WG-11) caused the highest mortality, though it was not statistically different from *H. bacteriophora* + *M. anisopliae* (WG-02) (Figure 2A).

Additive interactions were observed in all the combinations. In pupae, single applications produced lower mortality compared to combined applications (*F* = 32.6, df = 7, 47, *p* < 0.01, Table 2). Again, the combination of *H. bacteriophora* + *B. bassiana* (WG-11) caused the highest mortality, though it was not statistically different from *H. bacteriophora* + *M. anisopliae* (WG-02) (Figure 2B) and interactions were additive (Table 2). In second instar larvae, combined treatments produced significantly higher mortality compared to single applications of each agent (*F* = 19.8, df = 7, 47, *p* < 0.01). The highest morality was observed with *H. bacteriophora* + *B. bassiana* (WG-11), though the treatment was not different from *H. bacteriophora* + *M. anisopliae* (WG-02) (Figure 2C; Table 2).

### 3.3. Plant Potted Bioassay in Geenhouse

The combined treatment applications produced a lower number of emerging adults compared to single treatments at 1 (*F* = 63.35, df = 7, 47, *p* < 0.01), 2 (*F* = 54.87, df = 7, 47, *p* < 0.01), 3 (*F* = 117.35, df = 7, 47, *p* < 0.01), 4 (*F* = 68.86, df = 7, 47, *p* < 0.01), 5 (*F* = 89.36, df = 7, 47, *p* < 0.01), 6 (*F* = 59.97, df = 7, 47, *p* < 0.01), and 7 (*F* = 36.19, df = 7, 47, *p* < 0.01) days after adult emergence began (Table 3). Among all the day intervals, the lowest number of adults emerged from the *H. bacteriophora* + *B. bassiana* (WG-11) treatment, followed by *H. bacteriophora* + *M. anisopliae* (WG-02), though they were not statistically different from each other among all the day intervals. The highest number of adults emerged on the first day, while reductions in adult emergence were observed in subsequent days. From day five onward, no adult emergence was observed among the combined application treatments (Table 3).

### 3.4. Field Trials

During the first year, 2017–2018, at 1 day before application (DBA), no differences (*F* = 3.12, df = 8, 53, *p* = 0.007) were observed among the number of larvae found per plant between the different treatments, and all plots looked similar. The combined applications of both agents resulted in lower numbers of larvae compared to the single treatments, and the treatment effects became more pronounced with the passage of time, as indicated at 3 (*F* = 10.70, df = 8, 53, *p* < 0.01), 7 (*F* = 20.76, df = 8, 53, *p* < 0.01), 11 (*F* = 93.35, df = 8, 53, *p* < 0.01), and 15 (*F* = 135.7, df = 8, 53, *p* < 0.01) days post-application (DPA) (Table 4). *Heterorhabditis bacteriophora* + *B. bassiana* (WG-11) produced lower numbers of individuals, but it was not statistically different from *H. bacteriophora* + *M. anisopliae* (WG-02) (except at 11 DPA). For adults, at 1 DBA, no differences (*F* = 2.48, df = 8, 53, *p* = 0.024) were observed among the number of adults found per plant between the different treatments. Similar to larvae, the combination treatments produced significantly lower numbers of adults compared to single applications, and treatment effects became more pronounced over time, as observed in 3 (*F* = 16.48, df = 8, 53, *p* < 0.01), 7 (*F* = 41.71, df = 8, 53, *p* < 0.01), 11 (*F* = 97.66, df = 8, 53, *p* < 0.01), and 15 (*F* = 112.08, df = 8, 53, *p* < 0.01) days. Among all the daily intervals, *H. bacteriophora* + *B. bassiana* (WG-11) exhibited lower numbers of adults emerged, but the difference was not significantly separated from the treatment *H. bacteriophora* + *M. anisopliae* (WG-02) (Table 4).

During the second year, 2018–2019, numerically higher numbers of thrips were observed compared to the first year, 2017–2018. Significantly lower numbers of larvae in treatments were observed at 1 DBA (*F* = 4.54; df = 8, 53; *p* < 0.01), 3 DPA (*F* = 3.42; df = 8, 53; *p* < 0.01), 7 DPA (*F* = 29.92; df = 8, 53; *p* < 0.01), 11 DPA (*F* = 50.30; df = 8, 53; *p* < 0.01), and 15 DPA (*F* = 137.84; df = 8, 53; *p* < 0.01), compared to the control (Table 5). The number of larvae captured per plant decreased with the passage of time in the treatment groups while an increasing trend in larvae was observed in control plots. Until 3 DPA, no differences were observed among treatments. From 7 to 15 DPA, the treatment *H. bacteriophora* + *B. bassiana* (WG-11) produced lower adults compared to *H. bacteriophora* + *M. anisopliae* (WG-02). In the case of adults, no significant differences among treatments were observed at 1 DBA (*F* = 2.23; df = 8, 53; *p* = 0.04) and 3 DPA (*F* = 2.32; df = 8, 53; *p* = 0.03), while at 7 DPA (*F* = 8.27; df = 8, 53; *p* < 0.01), 11 DPA (*F* = 15.34; df = 8, 53; *p* < 0.01), and 15 DPA (*F* = 80.27; df = 8, 53; *p* < 0.01), treatments significantly produced fewer adults compared to the control (Table 5).

All the treatments significantly increased the leaf length (*F* = 13.4; df = 8, 53; *p* < 0.01 for 2017–2018 and *F* = 40.3; df = 8, 53; *p* < 0.01 for 2018–2019), leaf diameter (*F* = 12.7; df = 8, 53; *p* < 0.01 for 2017–2018; *F* = 26.0; df = 8, 53; *p* < 0.01 for 2018–2019), total number of leaves produced per plant (*F* = 12.6; df = 8, 53; *p* < 0.01 for 2017–2018; *F* = 16.2; df = 8, 53; *p* < 0.01 for 2018–2019), neck diameter (*F* = 1.58; df = 8, 53; *p* = 0.15 for 2017–2018; *F* = 1.11; df = 8, 53; *p* = 0.37 for 2018–2019), bulb diameter (*F* = 4.50; df = 8, 53; *p* < 0.01 for 2017–2018; *F* = 3.83; df = 8, 53; *p* < 0.01 for 2018–2019), number of rings produced per bulb (*F* = 12.5; df = 8, 53; *p* < 0.01 for 2017–2018; *F* = 20.8; df = 8, 53; *p* < 0.01 for 2018–2019), dry matter of plant (*F* = 9.75; df = 8, 53; *p* < 0.01 for 2017–2018; *F* = 8.14; df = 8, 53; *p* < 0.01 for 2018–2019), and total yield per plant (*F* = 38.3; df = 8, 53; *p* < 0.01 for 2017–2018; *F* = 48.1; df = 8, 53; *p* < 0.01 for 2018–2019) (Table 6). For both seasons, the combined application of both agents increased plant growth more, compared with single applications of each agent. In single applications, fungal treatments performed better than nematode applications (Table 6).

## 4. Discussion

Our results revealed that *B. bassiana* (WG-11), *Metarhizium anisopliae* (WG-02), *H. bacteriophora*, and *S. feltiae* nematodes were pathogenic to pre-pupae, pupae, and late second instar larvae of *T. tabaci*. In the laboratory bioassays (small cup and potted), combined application of *H. bacteriophora* + *B. bassiana* (WG-11) and *H. bacteriophora* + *M. anisopliae* (WG-02) exhibited higher mortalities than other treatments. The only synergism observed was in the small cup bioassay between *H. bacteriophora* + *B. bassiana* (WG-11), whereas in the rest of the combinations, additive interactions were produced. In the field trials, significantly lower numbers of larvae and adults in 2017–18 and 2018–19 were observed in the *H. bacteriophora* + *B. bassiana* (WG-11) and *H. bacteriophora* + *M. anisopliae* (WG-02) treatments compared to others. Along with damage reduction, treatments also resulted in increased plant growth, with better plant development observed in the combination of treatments.

Regarding the effects of single treatments, fungi were more effective than nematodes in our study. Moreover, *B. bassiana* (WG-11) exhibited higher mortality rates compared to *M. anisopliae* (WG-02). Previous studies have also reported high levels of virulence when testing various strains of *B. bassiana* to *T. tabaci* [39]. *Metarhizium anisopliae* has also been shown to exhibit efficacy to this pest [37]. Greater mortality of *T. tabaci* was observed when *B. bassiana* was applied as a foliar application and soil drenching of neem extract was applied [38]. Previously, no study was available on the efficacy of entomopathogenic fungi against the soil dwelling stages of *T. tabaci*. Thus, this is first study in which the effectiveness of EPF against soil dwelling stages of *T. tabaci* was tested.

In this study, a high level of efficacy of *H. bacteriophora* and *S. feltiae* against soil dwelling stages of *T. tabaci* was observed. Efficacy of *S. feltiae* and *H. bacteriophora* against *T. tabaci* was reported previously by [30]. *Heterorhabditis bacteriophora* demonstrated higher control of nymph and adult stages of *T. tabaci* under laboratory condition [57]. Other studies indicate that EPNs can be effective against another thrips species, *F. occidental* [32,58].

The goal of using the integrated techniques is to achieve a higher level of precision, accuracy, and reliability. The application of two different bio-control agents to the same pest may enhance the results by attacking independently at different points of vulnerability in the host. However, competitive factors among the control agents may lead to antagonistic effects. An additive interaction is considered when two biological control agents act independently from each other, while antagonistic or synergistic interactions make the combination more or less effective than additive interactions [47]. Koppenhöfer and Grewal (2005) [47] and Ansari et al. [44] suggested that, when fungi and nematodes are applied at same time, their interactions have additive effects on insect mortality because both agents act independently, but it depends on the particular combinations of pathogens and host species. In the current study, combined infection of EPF and EPNs resulted in additive and, in one case, synergistic interactions. This was the first study on combining EPF and EPNs for *T. tabaci* control. Targeting another thrips species, the combined application of *M. anisopliae* and *S. carpocapsae* reduced the adult emergence of *F. occidentalis* up to 74% [50]. Additionally, positive interactions between EPNs and EPF have been cited in other pest systems [44,49,59,60,61]. For example, Ansari et al. [45] found synergistic interactions between *M. anisopliae* and EPNs against 3rd instar larvae of the black vine weevil *Otiorhynchus sulcatus* Fabricius (Coleoptera: Curculionidae) when applied at the same time. In contrast, Correa-Cuadros et al. [62] reported that mortality due to EPF and EPNs applied individually caused higher mortality (antagonism). Antagonistic effects were also found in some other studies by [48,56,59], which are contradictory to our results. Antagonism can be caused by various levels of competition between the two organisms for nutrition, growth, oxygen demand, or via the production of metabolites that adversely affect the other control agent [44,45,48,63]. The nature of interaction between two microbial agents (additivity, synergy, or antagonism) can depend on the rate and timing of application [46,55]; conceivably the timing and rates of the EPF–EPN combinations used in this study could be manipulated to produce synergy in a consistent manner, but that will require further research.

The basis for the differential interactions that we observed among the EPN–EPF combinations is not clear. EPF and EPN (as well as the symbiotic bacteria associated with the EPNs) produce toxins that may be antagonistic to the competing microbial agent [64]. On the other hand, these same toxins that weaken the insect may also facilitate infection by the other microbial agent, leading to synergy [47]. The particular toxins or other factors involved in driving EPN–EPF interactions may be another area of fruitful research.

## 5. Conclusions

The combined use and interaction effects of entomopathogenic fungi and nematodes were evaluated as innovative alternatives to combat *T. tabaci*. Our results indicate that certain microbial combinations (such as *H. bacteriophora* combined with *B. bassiana* or *M. anisopliae*) could lead to improved management of the target pest. In contrast, single treatment applications of EPF or EPNs did not appear to be as promising as combined applications; efficacy was clearly lower when the microbial agents were applied alone. The combined applications that exhibited additivity and synergy should be compatible as tank-mixes and may result in enhanced biocontrol efficacy in the field. Both EPN and EPF are commercially applied in various systems and, thus, their adoption to *T. tabaci* control should be straight forward. Moreover, combined application of EPN–EPF for *T. tabaci* control would fit naturally into IPM strategies that incorporate multi-stage tactics against the pest. However, from a practical side, combination of both entomopathogens will depend upon the effectiveness of combination, and their costs in relation to competition with synthetic insecticides. Additional research is required to determine the optimization and feasibility of combined microbial treatments for control of *T. tabaci* under field conditions.

## Figures and Tables

**Table 1:** Interactions between *Heterorhabditis bacteriophora* (Hb), *Steinernema feltiae* (Sf), *Beauveria bassiana* (WG-11), and *Metarhizium anisopliae* (WG-02) against late second instar larvae, pre-pupae, and pupae of *Thrips tabaci* in a laboratory small cup bioassay.

Stage	Treatments	Observed Mortality (%)	Expected Mortality (%)	Chi Square	Interaction
Pre-pupae	Hb + WG-11	94.73	77.10	4.03	Synergism
	Hb + WG-02	85.96	74.23	1.85	Additive
	Sf + WG-11	77.19	74.15	0.12	Additive
	Sf + WG-02	66.66	70.91	0.25	Additive
Pupae	Hb + WG-11	82.45	66.75	3.69	Additive
	Hb + WG-02	73.68	62.32	2.06	Additive
	Sf + WG-11	64.91	63.06	0.05	Additive
	Sf + WG-02	59.64	58.14	0.03	Additive
Late second instar larvae	Hb + WG-11	68.42	55.83	2.83	Additive
	Hb + WG-02	63.15	52.04	2.37	Additive
	Sf + WG-11	56.14	52.60	0.23	Additive
	Sf + WG-02	49.12	48.53	0.007	Additive

**Table 2:** Interactions between *Heterorhabditis bacteriophora* (Hb), *Steinernema feltiae* (Sf), *Beauveria bassiana* (WG-11), and *Metarhizium anisopliae* (WG-02) against late second instar larvae, pre-pupae, and pupae of *Thrips tabaci* in a laboratory potted soil bioassay.

Stage	Treatments	Observed Mortality (%)	Expected Mortality (%)	Chi Square	Interaction
Pre-pupae	Hb + WG-11	97.36	83.91	2.15	Additive
	Hb + WG-02	85.96	80.53	0.36	Additive
	Sf + WG-11	81.57	80.99	0.004	Additive
	Sf + WG-02	74.56	76.99	0.07	Additive
Pupae	Hb + WG-11	90.43	75.55	2.93	Additive
	Hb + WG-02	79.13	72.78	0.55	Additive
	Sf + WG-11	73.91	69.94	0.22	Additive
	Sf + WG-02	70.43	66.54	0.22	Additive
Late second instar larvae	Hb + WG-11	78.94	64.21	3.37	Additive
	Hb + WG-02	71.92	60.75	2.05	Additive
	Sf + WG-11	65.78	62.77	0.14	Additive
	Sf + WG-02	63.15	59.18	0.26	Additive

**Table 3:** Mean (± SE) adult emergence of *Thrips tabaci* at 1, 2, 3, 4, 5, 6, and 7 days when treated with Hb = *Heterorhabditis bacteriophora*, Sf = *Steinernema feltiae*, WG-11 = *Beauveria bassiana*, WG-02 = *Metarhizium anisopliae* (WG-02), Hb + WG-11 = *H. bacteriophora* + *B. bassiana*, Hb + WG-02 = *H. bacteriophora* + *M. anisopliae*, Sf + WG-11 = *S. feltiae* + *B. bassiana*, Sf + WG-02 = *S. feltiae* + *M. anisopliae,* control = water only in a potted plant bioassay under greenhouse conditions. Different letters in each column indicate statistical significance (*p* < 0.05; Tukey’s test).

Treatments	1 Day	2 Day	3 Day	4 Day	5 Day	6 Day	7 Day
Hb	28.33 ± 1.28 ^bc^	20.50 ± 1.56 ^bc^	13.16 ± 1.19 ^c^	10.16 ± 0.70 ^c^	7.16 ± 0.79 ^c^	3.83 ± 0.60 ^c^	1.66 ± 0.33 ^b^
Sf	32.50 ± 1.11 ^ab^	25.16 ± 1.07 ^ab^	17.50 ± 1.43 ^b^	14.50 ± 1.25 ^b^	10.33 ± 0.71 ^b^	6.50 ± 0.76 ^b^	0.00 ± 0.00 ^c^
WG-11	19.66 ± 1.45 ^de^	12.50 ± 1.68 ^de^	8.33 ± 0.55 ^de^	6.66 ± 0.88 ^cde^	3.66 ± 0.49 ^d^	1.66 ± 0.33 ^cd^	0.00 ± 0.00 ^c^
WG-22	24.83 ± 1.92 ^cd^	16.33 ± 1.80 ^cd^	10.66 ± 0.88 ^cd^	7.83 ± 0.87 ^cd^	4.16 ± 0.30 ^cd^	1.33 ± 0.42 ^d^	0.00 ± 0.00 ^c^
Hb + WG-11	8.66 ± 0.55 ^g^	5.00 ± 0.73 ^f^	2.50 ± 0.42 ^f^	1.66 ± 0.33 ^f^	0.00 ± 0.00 ^e^	0.00 ± 0.00 ^d^	0.00 ± 0.00 ^c^
Hb + WG-22	10.33 ± 0.49 ^fg^	5.33 ± 0.71 ^f^	2.66 ± 0.33 ^f^	2.50 ± 0.42 ^ef^	0.00 ± 0.00 ^e^	0.00 ± 0.00 ^d^	0.00 ± 0.00 ^c^
Sf + WG-11	14.83 ± 1.10 ^ef^	7.16 ± 0.65 ^ef^	5.83 ± 0.60 ^ef^	3.66 ± 0.33 ^def^	0.00 ± 0.00 ^e^	0.00 ± 0.00 ^d^	0.00 ± 0.00 ^c^
Sf + WG-22	17.83 ± 1.24 ^e^	9.16 ± 0.83 ^ef^	8.16 ± 0.60 ^de^	4.16 ± 0.40 ^def^	0.00 ± 0.00 ^e^	0.00 ± 0.00 ^d^	0.00 ± 0.00 ^c^
Control	37.33 ± 1.11 ^a^	29.16 ± 1.16 ^a^	31.00 ± 1.06 ^a^	25.50 ± 1.64 ^a^	18.83 ± 1.57 ^a^	11.66 ± 0.98 ^a^	4.83 ± 0.74 ^a^
Df	8, 53	8, 53	8, 53	8, 53	8, 53	8, 53	8, 53
*F*	63.35	54.87	117.35	68.86	89.36	59.97	36.19
*P*	<0.01	<0.01	<0.01	<0.01	<0.01	<0.01	<0.01

**Table 4:** Mean (±SE) adult emergence of *Thrips tabaci* at 1 day before application (DBA), and 1, 3, 7, 11, and 15 days post-application (DPA) when treated with Hb = *Heterorhabditis bacteriophora*, Sf = *Steinernema feltiae*, WG-11 = *Beauveria bassiana* (WG-11), WG-22 = *Metarhizium anisopliae* (WG-02), Hb + WG-11 = *H. bacteriophora* + *B. bassiana*, Hb + WG-02 = *H. bacteriophora* + *M. anisopliae*, Sf + WG-11 = *S. feltiae* + *B. bassiana*, Sf + WG-02 = *S. feltiae* + *M. anisopliae,* control = water only under field conditions during 2017–2018. Different letters in each column indicate statistical significance (*p* < 0.05; Tukey’s test).

Stage	Treatments	1 DBA	3 DPA	7 DPA	11 DPA	15 DPA
Larvae	Hb	48.85 ± 1.30 ^ab^	44.53 ± 1.75 ^ab^	38.51 ± 2.00 ^bc^	32.75 ± 1.03 ^b^	25.03 ± 0.99 ^b^
	Sf	49.13 ± 1.72 ^a^	45.60 ± 1.69 ^a^	40.11 ± 1.85 ^b^	34.05 ± 1.18 ^b^	27.21 ± 1.67 ^b^
	WG-11	48.38 ± 1.34 ^ab^	44.48 ± 1.91 ^ab^	37.51 ± 1.74 ^bc^	30.31 ± 1.36 ^bc^	22.61 ± 1.24 ^bc^
	WG-02	46.46 ± 1.37 ^ab^	42.05 ± 1.46 ^abc^	36.51 ± 1.16 ^bc^	27.16 ± 0.98 ^cd^	19.41 ± 1.13 ^cd^
	Hb + WG-11	43.06 ± 1.42 ^ab^	33.10 ± 1.29 ^d^	24.58 ± 1.33 ^e^	11.86 ± 0.88 ^g^	2.86 ± 0.42 ^g^
	Hb + WG-02	49.00 ± 1.58 ^a^	36.35 ± 1.31 ^cd^	28.86 ± 0.96 ^de^	17.75 ± 0.80 ^f^	7.13 ± 0.77 ^fg^
	Sf + WG-11	42.05 ± 0.81 ^b^	38.16 ± 1.57 ^bcd^	32.11 ± 1.13 ^cd^	20.56 ± 0.87 ^ef^	11.65 ± 0.78 ^ef^
	Sf + WG-02	47.45 ± 1.39 ^ab^	41.25 ± 1.88 ^abc^	35.66 ± 1.63 ^bcd^	23.03 ± 0.92 ^de^	14.28 ± 0.93 ^de^
	Control	46.38 ± 1.71 ^ab^	47.10 ± 1.41 ^a^	47.80 ± 1.33 ^a^	48.70 ± 1.42 ^a^	49.01 ± 1.64 ^a^
	df	8, 53	8, 53	8, 53	8, 53	8, 53
	*F*	3.12	10.70	20.76	93.35	135.17
	*p*	0.0078	<0.01	<0.01	<0.01	<0.01
Adult	Hb	33.03 ± 0.87 ^ab^	31.06 ± 0.70 ^ab^	26.50 ± 0.95 ^b^	21.70 ± 0.75 ^b^	15.33 ± 1.31 ^bc^
	Sf	32.30 ± 1.01 ^ab^	31.01 ± 0.27 ^ab^	27.98 ± 0.86 ^b^	22.01 ± 0.87 ^b^	17.90 ± 1.42 ^b^
	WG-11	32.71 ± 1.23 ^ab^	29.61 ± 1.23 ^bc^	23.85 ± 1.13 ^bc^	16.13 ± 1.04 ^cd^	12.63 ± 1.33 ^cd^
	WG-02	34.26 ± 0.90 ^ab^	30.76 ± 0.76 ^ab^	25.90 ± 0.97 ^b^	18.15 ± 1.07 ^bc^	13.43 ± 1.30 ^bcd^
	Hb + WG-11	33.48 ± 0.75 ^ab^	22.38 ± 0.81 ^e^	13.46 ± 0.96 ^f^	5.51 ± 0.63 ^f^	1.68 ± 0.19 ^f^
	Hb + WG-02	35.01 ± 0.75 ^a^	24.11 ± 1.17 ^de^	16.08 ± 1.07 ^ef^	8.18 ± 0.59 ^ef^	3.05 ± 0.35 ^f^
	Sf + WG-11	31.41 ± 0.77 ^ab^	25.30 ± 1.09 ^cde^	19.08 ± 1.87 ^de^	12.15 ± 0.92 ^de^	7.96 ± 0.98 ^ef^
	Sf + WG-02	34.33 ± 1.23 ^ab^	27.35 ± 1.33 ^bcd^	21.23 ± 0.91 ^cd^	14.21 ± 1.19 ^cd^	9.46 ± 0.68 ^de^
	Control	30.08 ± 0.74 ^b^	34.10 ± 0.61 ^a^	34.60 ± 0.50 ^a^	36.05 ± 0.69 ^a^	36.96 ± 0.82 ^a^
	df	8, 53	8, 53	8, 53	8, 53	8, 53
	*F*	2.48	16.48	41.71	97.66	112.08
	*p*	0.0274	<0.01	<0.01	<0.01	<0.01

**Table 5:** Mean (± SE) adult and larvae of *Thrips tabaci* at 1 day before application (DBA), and 1, 3, 7, 11, and 15 days post-application (DPA) treatment when treated with Hb = *Heterorhabditis bacteriophora*, Sf = *Steinernema feltiae*, WG-11 = *Beauveria bassiana* (WG-11), WG-22 = *Metarhizium anisopliae* (WG-02), Hb + WG-11 = *H. bacteriophora* + *B. bassiana*, Hb + WG-02 = *H. bacteriophora* + *M. anisopliae*, Sf + WG-11 = *S. feltiae* + *B. bassiana*, Sf + WG-02 = *S. feltiae* + *M. anisopliae,* control = water only under field conditions during 2018–2019. Different letters in each column indicate statistical significance (*p* < 0.05; Tukey’s test).

Stage	Treatments	1 DBA	3 DPA	7 DPA	11 DPA	15 DPA
Larvae	Hb	53.61 ± 1.85 ^abc^	50.08 ± 2.71 ^a^	43.53 ± 1.32 ^bc^	37.01 ± 1.74 ^b^	26.63 ± 1.49 ^bc^
	Sf	55.21 ± 1.88 ^ab^	51.10 ± 3.15 ^a^	45.36 ± 1.82 ^b^	40.06 ± 2.12 ^b^	31.41 ± 1.94 ^b^
	WG-11	57.25 ± 2.06 ^a^	48.40 ± 2.55 ^ab^	41.21 ± 1.08 ^bcd^	32.81 ± 1.89 ^bc^	19.95 ± 1.10 ^de^
	WG-02	52.06 ± 1.48 ^abc^	49.56 ± 2.01 ^a^	43.18 ± 1.39 ^bc^	35.90 ± 2.10 ^b^	24.06 ± 1.18 ^cd^
	Hb + WG-11	48.91 ± 1.18 ^bc^	40.76 ± 1.59 ^b^	26.91 ± 2.13 ^f^	15.25 ± 1.14 ^e^	6.11 ± 0.90 ^h^
	Hb + WG-02	47.11 ± 1.50 ^c^	43.53 ± 1.62 ^ab^	29.11 ± 1.00 ^ef^	19.71 ± 1.68 ^de^	9.58 ± 0.30 ^gh^
	Sf + WG-11	51.10 ± 1.72 ^abc^	45.65 ± 1.04 ^ab^	35.78 ± 1.63 ^de^	23.10 ± 1.12 ^d^	13.11 ± 0.84 ^fg^
	Sf + WG-02	50.13 ± 0.58 ^abc^	46.73 ± 1.47 ^ab^	38.21 ± 1.58 ^cd^	26.23 ± 1.54 ^cd^	17.36 ± 1.73 ^ef^
	Control	48.61 ± 1.15 ^bc^	50.45 ± 1.55 ^a^	52.35 ± 1.32 ^a^	53.98 ± 1.41 ^a^	54.58 ± 1.37 ^a^
	df	8, 53	8, 53	8, 53	8, 53	8, 53
	*F*	4.54	3.42	29.92	50.30	137.84
	*p*	<0.01	<0.01	<0.01	<0.01	<0.01
Adult	Hb	36.61 ± 1.84 ^a^	34.70 ± 1.74 ^ab^	28.46 ± 2.73 ^abc^	25.30 ± 2.10 ^bc^	14.23 ± 1.48 ^bc^
	Sf	34.08 ± 1.84 ^ab^	32.51 ± 1.78 ^ab^	29.65 ± 2.53 ^ab^	26.48 ± 2.47 ^b^	17.08 ± 1.40 ^b^
	WG-11	35.06 ± 1.89 ^ab^	30.06 ± 2.17 ^ab^	25.48 ± 1.62 ^bcd^	23.30 ± 1.89 ^bcd^	11.08 ± 0.84 ^cd^
	WG-02	32.78 ± 2.06 ^ab^	31.13 ± 2.24 ^ab^	27.43 ± 2.00 ^abc^	24.45 ± 1.97 ^bc^	13.70 ± 0.70 ^bc^
	Hb + WG-11	30.68 ± 2.62 ^ab^	25.83 ± 2.12 ^b^	17.86 ± 1.52 ^d^	12.78 ± 1.35 ^e^	3.80 ± 0.42 ^e^
	Hb + WG-02	32.13 ± 1.87 ^ab^	28.61 ± 2.00 ^ab^	20.93 ± 1.56 ^cd^	15.31 ± 1.19 ^de^	7.73 ± 0.54 ^de^
	Sf + WG-11	33.31 ± 2.08 ^ab^	29.51 ± 1.64 ^ab^	22.68 ± 0.85 ^bcd^	17.63 ± 1.73 ^cde^	8.78 ± 0.53 ^d^
	Sf + WG-02	27.55 ± 2.37 ^b^	30.93 ± 1.52 ^ab^	24.91 ± 1.88 ^bcd^	20.08 ± 1.96 ^bcde^	11.68 ± 0.93 ^cd^
	Control	35.33 ± 1.95 ^ab^	35.83 ± 2.23 ^a^	35.96 ± 2.26 ^a^	36.13 ± 1.90 ^a^	36.90 ± 1.91 ^a^
	df	8, 53	8, 53	8, 53	8, 53	8, 53
	*F*	2.23	2.32	8.27	15.34	80.27
	*p*	0.04	0.03	<0.01	<0.01	<0.01

**Table 6:** Effect of different treatments Hb = Heterorhabditis bacteriophora, Sf = Steinernema feltiae, WG-11 = Beauveria bassiana (WG-11), WG-02 = Metarhizium anisopliae (WG-02), Hb + WG-11 = H. bacteriophora + B. bassiana, Hb + WG-02 = H. bacteriophora + M. anisopliae, Sf + WG-11 = S. feltiae + B. bassiana, Sf + WG-02 = S. feltiae + M. anisopliae, control = water only under field conditions during 2017–2018 and 2018–2019 on various attributes of onion plants. Different letters in each column indicate statistical significance (*p* < 0.05; Tukey’s test).

Season	Treatment	Leaves Length (cm)	Leaf Weight (g)	No. of Leaves	Neck Diameter (cm)	Bulb Diameter (cm)	No. of Rings/Bulb	Dry Matter (%)	Yield/Plant (g)
2017–2018	Hb	40.00 ± 0.52 ^c^	15.45 ± 0.45 ^de^	12.33 ± 0.42 ^cd^	0.75 ± 0.05 ^a^	5.78 ± 0.34 ^bc^	65.01 ± 0.41 ^cd^	13.00 ± 0.48 ^c^	75.11 ± 0.59 ^ef^
	Sf	39.35 ± 0.39 ^c^	15.41 ± 0.37 ^de^	11.21 ± 0.40 ^d^	0.73 ± 0.03 ^a^	5.45 ± 0.37 ^c^	65.48 ± 0.44 ^cd^	12.43 ± 0.43 ^c^	74.15 ± 0.46 ^ef^
	WG-11	41.16 ± 0.44 ^bc^	16.43 ± 0.48 ^bcde^	13.30 ± 0.50 ^bc^	0.78 ± 0.04 ^a^	6.13 ± 0.47 ^abc^	66.25 ± 0.40 ^bcd^	14.06 ± 0.35 ^abc^	76.01 ± 0.47 ^de^
	WG-02	41.28 ± 0.51 ^bc^	16.00 ± 0.45 ^cde^	12.01 ± 0.45 ^cd^	0.75 ± 0.04 ^a^	6.03 ± 0.55 ^abc^	65.01 ± 0.45 ^cd^	13.18 ± 0.45 ^bc^	75.05 ± 0.49 ^ef^
	Hb + WG-11	44.31 ± 0.53 ^a^	19.25 ± 0.42 ^a^	15.01 ± 0.46 ^ab^	0.88 ± 0.04 ^a^	7.95 ± 0.45 ^a^	69.55 ± 0.48 ^a^	16.01 ± 0.51 ^a^	82.13 ± 0.41 ^a^
	Hb + WG-02	43.30 ± 0.47 ^ab^	18.05 ± 0.51 ^abc^	15.53 ± 0.46 ^a^	0.85 ± 0.04 ^a^	7.63 ± 0.43 ^ab^	68.00 ± 0.40 ^ab^	15.20 ± 0.40 ^ab^	80.01 ± 0.48 ^ab^
	Sf + WG-11	42.23 ± 0.51 ^ab^	18.46 ± 0.49 ^ab^	14.01 ± 0.48 ^abc^	0.83 ± 0.04 ^a^	6.93 ± 0.50 ^abc^	67.00 ± 0.49 ^bc^	15.56 ± 0.49 ^a^	78.40 ± 0.41 ^bc^
	Sf + WG-02	42.40 ± 0.47 ^ab^	17.01 ± 0.41 ^bcd^	13.10 ± 0.41 ^bcd^	0.80 ± 0.04 ^a^	6.33 ± 0.51 ^abc^	66.38 ± 0.46 ^bcd^	14.15 ± 0.35 ^abc^	77.40 ± 0.42 ^cd^
	Control	39.28 ± 0.39 ^c^	14.36 ± 0.41 ^e^	11.06 ± 0.37 ^d^	0.71 ± 0.04 ^a^	5.06 ± 0.40 ^c^	64.60 ± 0.51 ^d^	12.25 ± 0.44 ^c^	73.36 ± 0.41 ^f^
	df	8, 53	8, 53	8, 53	8, 53	8, 53	8, 53	8, 53	8, 53
	*F*	13.4	12.7	12.6	1.58	4.50	12.5	9.75	38.3
	*p*	<0.01	<0.01	<0.01	0.15	<0.01	<0.01	<0.01	<0.01
2018–2019	Hb	38.05 ± 0.62 ^de^	14.53 ± 0.42 ^cd^	11.13 ± 0.37 ^bc^	0.71 ± 0.04 ^a^	5.60 ± 0.40 ^abc^	63.65 ± 0.55 ^def^	13.26 ± 0.44 ^cde^	73.21 ± 0.47 ^ef^
	Sf	37.08 ± 0.39 ^e^	14.25 ± 0.45 ^cd^	10.48 ± 0.42 ^c^	0.70 ± 0.03 ^a^	5.26 ± 0.38 ^bc^	62.93 ± 0.73 ^ef^	12.45 ± 0.43 ^de^	71.25 ± 0.39 ^fg^
	WG-11	41.38 ± 0.43 ^bc^	16.01 ± 0.36 ^bc^	11.25 ± 0.47 ^bc^	0.75 ± 0.05 ^a^	5.98 ± 0.34 ^abc^	65.41 ± 0.45 ^bcd^	13.80 ± 0.46 ^abcde^	75.00 ± 0.51 ^cde^
	WG-02	40.05 ± 0.38 ^cd^	15.03 ± 0.42 ^c^	12.20 ± 0.52 ^bc^	0.73 ± 0.05 ^a^	5.80 ± 0.40 ^abc^	64.81 ± 0.42 ^cde^	13.61 ± 0.47 ^bcde^	74.15 ± 0.36 ^de^
	Hb + WG-11	44.38 ± 0.46 ^a^	19.01 ± 0.37 ^a^	15.10 ± 0.52 ^a^	0.83 ± 0.04 ^a^	7.45 ± 0.45 ^a^	68.76 ± 0.52 ^a^	15.75 ± 0.41 ^a^	80.26 ± 0.42 ^a^
	Hb + WG-02	43.15 ± 0.38 ^ab^	18.20 ± 0.44 ^a^	14.38 ± 0.41 ^a^	0.80 ± 0.03 ^a^	7.28 ± 0.44 ^ab^	68.11 ± 0.46 ^a^	15.33 ± 0.35 ^ab^	78.10 ± 0.40 ^b^
	Sf + WG-11	43.53 ± 0.40 ^a^	18.51 ± 0.47 ^a^	14.55 ± 0.36 ^a^	0.78 ± 0.04 ^a^	6.73 ± 0.48 ^abc^	67.36 ± 0.43 ^ab^	14.85 ± 0.44 ^abc^	77.03 ± 0.48 ^bc^
	Sf + WG-02	42.46 ± 0.45 ^ab^	17.38 ± 0.37 ^ab^	13.01 ± 0.49 ^ab^	0.76 ± 0.04 ^a^	6.45 ± 0.46 ^abc^	66.88 ± 0.43 ^abc^	14.16 ± 0.40 ^abcd^	76.18 ± 0.41 ^bcd^
	Control	37.26 ± 0.38 ^e^	13.05 ± 0.42 ^d^	10.45 ± 0.44 ^c^	0.68 ± 0.03 ^a^	5.03 ± 0.51 ^c^	62.40 ± 0.47 ^f^	12.15 ± 0.43 ^e^	71.03 ± 0.49 ^g^
	df	8, 53	8, 53	8, 53	8, 53	8, 53	8, 53	8, 53	8, 53
	*F*	40.3	26.0	16.2	1.11	3.83	20.8	8.14	48.1
	*p*	<0.01	<0.01	<0.01	0.37	<0.01	<0.01	<0.01	<0.01

## Data Availability

Data is contained within the article.
